# Identification of a Five Immune Term Signature for Prognosis and Therapy Options (Immunotherapy versus Targeted Therapy) for Patients with Hepatocellular Carcinoma

**DOI:** 10.1155/2023/8958962

**Published:** 2023-02-02

**Authors:** Xiaoyun Bin, Zongjiang Luo, Jianchu Wang, Sufang Zhou

**Affiliations:** ^1^Department of Biochemistry and Molecular Biology, School of Basic Medical Sciences, Guangxi Medical University, Nanning, Guangxi 530021, China; ^2^Department of Hepatobiliary Surgery, Affiliated Hospital of Youjiang Medical University for Nationalities, Guangxi 533000, China; ^3^Key Laboratory of Longevity and Aging-Related Diseases of Chinese Ministry of Education, Center for Translational Medicine, Guangxi Medical University, Nanning, Guangxi, China

## Abstract

**Background:**

Immune microenvironment implicated in liver cancer development. Nevertheless, previous studies have not fully investigated the immune microenvironment in liver cancer.

**Methods:**

The open-access data used for analysis were obtained from The Cancer Genome Atlas (TCGA-LIHC) and the International Cancer Genome Consortium databases (ICGC-JP and ICGC-FR). R program was employed to analyze all the data statistically.

**Results:**

First, the TCGA-LIHC, ICGC-FR, and ICGC-JP cohorts were selected for our analysis, which were merged into a combined cohort. Then, we quantified 53 immune terms in this combined cohort with large populations using the ssGSEA algorithm. Next, a prognostic approach was established based on five immune principles (CORE.SERUM.RESPONSE.UP, angiogenesis, CD8.T.cells, Th2.cells, and B.cells) was established, which showed great prognostic prediction efficiency. Clinical correlation analysis demonstrated that high-risk patients could reveal higher progressive clinical features. Next, to examine the inherent biological variations in high- and low-risk patients, pathway enrichment tests were conducted. DNA repair, E2F targets, G2M checkpoints, HEDGEHOG signaling, mTORC1 signaling, and MYC target were positively correlated with the risk score. Examination of genomic instability revealed that high-risk patients may exhibit a higher tumor mutation burden score. Meanwhile, the risk score showed a strong positive correlation with the tumor stemness index. In addition, the Tumor Immune Dysfunction and Exclusion outcome indicated that high-risk patients could be higher responsive to immunotherapy, whereas low-risk patients may be higher responsive to Erlotinib. Finally, six characteristic genes DEPDC1, DEPDC1B, NGFR, CALCRL, PRR11, and TRIP13 were identified for risk group prediction.

**Conclusions:**

In summary, our study identified a signature as a useful tool to indicate prognosis and therapy options for liver cancer patients.

## 1. Introduction

Every year, around 840, 000 new cases of liver cancer are diagnosed and 780, 000 people lose their lives as a result of this disease. Hepatocellular carcinoma is the most common pathological subtype among all cases, accounting for 90% of primary liver cancer [1]. As a multifactorial and multicause disease, the genesis and progression of liver cancer are linked to numerous risk variables, such as genetics, lifestyle, and environmental aspects [2]. At present, surgery is still the first-line therapy option for liver cancer patients in the early stage [3]. However, considering its insidious symptoms, a substantial portion of patients have been in an advanced stage when first diagnosed, therefore, leading to loss of the appropriate timing of surgery [4]. Meanwhile, due to the characteristic of rapid progression and early metastasis, liver cancer patients tend to have a poor prognosis [2]. Therefore, it is important to find novel molecules and directions with implications for liver cancer diagnosis and therapy.

The tumor microenvironment (TME) significantly affects the biological process in cancer progression [5]. Immune cells and status are the essential components in TME. Different factors, such as cytokines, chemokines, and others, can help cancer cells shape their microenvironment to support their growth [6]. Meanwhile, the reprogramming of other cells surrounding cancer cells plays a decisive role in tumor survival and progression [7]. An example is that TME can enhance immunosuppressive M2 monocyte-derived macrophages by secreting cytokines such as IL-4, which allows the tumor to grow and progress because monocyte-derived macrophages can account for 50% of the tumor mass [8]. Also, through a CXCL13/CXCR5/NFB/p65/miR-934 positive feedback mechanism, Zhao et al. demonstrated that tumor-derived exosomal miR-934 might stimulate macrophage M2 polarizing action to increase liver metastasis of colorectal malignancy [9]. The crosstalk between immune factors and other systems can make TME more complex [10]. In TME, cancer cells showed different metabolic modes from normal cells, like the “Warburg” effect, which has a broader significance in regulating tumor immunity [10]. This energetic interaction involving tumor and immune cells results in metabolic conflict in the tumor ecosystem, restricts the number of nutrients, and causes microenvironmental acidity, which inhibits the activity of immune cells ^10^. In addition, metabolic reprogramming is essential for the maintenance of immune cell stability and body balance [11]. Presently, increasing studies have pointed out that metabolic reprogramming occurs in the mechanism of immune cell growth, development, and functional activity, which is crucial for immune reaction [12]. Nowadays, immunotherapy presents promising therapeutic effects in specific cancer populations. Cancer immune status can also influence the intensity and time of the anticancer reaction of immunotherapy [13]. Therefore, it is meaningful to explore the immune microenvironment in liver cancer.

Here, we firstly quantified 53 immune terms in a combined cohort with large populations using the ssGSEA algorithm. Then, a prognostic modeling was created based on five immunological elements (CORE.SERUM.RESPONSE.UP, angiogenesis, CD8.T.cells, Th2.T.cells, and B.cells), which showed great prognosis prediction efficiency. Analysis of clinical correlations revealed that high-risk patients may exhibit higher clinical characteristics progression. Subsequently, a pathway enrichment analysis was conducted to investigate intrinsic biological variations between high- and low-risk patients. Assessment of genomic instability revealed that high-risk individuals could possess a greater TMB score. The risk score showed a high positive correlation with tumor stemness index. In addition, the Tumor Immune Dysfunction and Exclusion (TIDE) outcome indicated that high-risk patients may show higher responsiveness to immunotherapy, whereas low-risk patients may show higher responsiveness to Erlotinib. Finally, six characteristic genes DEPDC1, DEPDC1B, NGFR, CALCRL, PRR11, and TRIP13 were identified for risk group prediction.

## 2. Methods

### 2.1. Data Acquisition

Open access data retrieval was carried out based on The Cancer Genome Atlas (TCGA; https://portal.gdc.cancer.gov/), Gene Expression Omnibus (GEO; https://www.ncbi.nlm.nih.gov/gds), and International Cancer Genome Consortium (ICGC; https://dcc.icgc.org/) databases (Access time: 2022/06/19). For the TCGA database, the expression profile information was obtained in a manner of “STAR-Counts” and then collated using the author's code. Clinical information was obtained in the “xml” form. For the GEO database, GSE14520 and GSE76427 were first included in the study for complete prognosis and transcriptional profiling data. After data quality evaluation, the GSE14520 was eliminated for the reason that the number of probes is less than 20,000; the GSE76427 was eliminated for a large number of missing values (NaN). For the ICGC, the ICGC-JP and ICGC-FR projects were included in the study. Sva package was applied for the data combination with batch effect reduction. First, by taking the logarithm, the order of magnitude of the data (TCGA and ICGC) reached the same range. Then, the combat function in sva package was used to detect and reduce the batch effect between different cohorts. Baseline information of the included patients was shown in Tables 1–3.

### 2.2. Immune Term Quantification

Immune elements were quantified using the single sample gene set enrichment analysis (ssGSEA) technique, which is an in-built algorithm of Gene Set Variation Analysis (GSVA) [14]. The reference immune terms set was obtained from the previous study, which was used to quantify the enrichment score of 53 immune terms [15]. The advantage of ssGSEA algorithm is the high freedom, in which you can quantify the enrichment score according to the given gene set. However, considering that gene sets can be freely defined, potential quality bias is inevitable.

### 2.3. Prognosis Analysis and Model Construction

Based on the assessment of 53 immunological elements from the ssGSEA algorithm, a univariate Cox regression analysis was conducted to discover the prognosis-related terms (*P* < 0.05). Afterward, the random survival forest variable hunting (RSFVH) method was processed to reduce the number of dimensions and filter genes. Finally, a multivariate Cox regression analysis was conducted for the development of a prognostic model. Kaplan-Meier (KM) analysis was employed to detect the best gene combination or final signature by analyzing log-rank *P* values. Each enrolled patient with complete prognosis and expression profile data was assigned a risk score with the formula of “Risk score = Terms A∗Coef A + Terms B∗Coef B + ⋯+Terms N∗Coef N” [15]. If the risk score was greater than the median value, the patients were classified into the high-risk or low-risk group, accordingly. ROC and KM survival curves were utilized to assess the accuracy of our model's prognostic projections. Univariate and multivariate Cox regression were also employed to validate the independence of the prediction model. The 1-, 3-, and 5-year survival can reflect the short-, medium-, and long-term prognosis of patients, and therefore, were selected as the time node in prognosis analysis.

### 2.4. Pathway Enrichment and Genomic Analysis

The GSVA and GSEA algorithms were utilized for pathway enrichment analysis [16]. Hallmark was used as the standard gene set for the GSVA algorithms, whereas metabolism-related gene sets (41 metabolism terms) were acquired from the website https://www.gsea-msigdb.org/. Standard gene sets for the GSEA algorithm were c2.cp.kegg.v7.5.1.symbols.gmt and c5.go.v7.5.1.symbols.gmt. The TCGA database was accessed using genomic mutation information, including the tumor mutation burden (TMB) and microsatellite instability (MSI) score. Based on the expression profile and utilizing the one-class logistic regression machine learning (OCLR) machine-learning technique, the tumor stemness index was computed [17].

### 2.5. Immunotherapy and Drug Sensitivity Analysis

Using the TIDE methodology [18], patients were evaluated for immunotherapy sensitivity. The parameter of “Cancer type” was set as “Other.” The parameter of “Previous immunotherapy” was set as “No.” The analysis of drug responsiveness was conducted using the database of Genomics of Drug Responsiveness in Cancer [19].

### 2.6. Feature Gene Identification

To identify the feature genes for the risk group, the LASSO regression and SVM-RFE (support vector machine recursive feature elimination) algorithm were applied to find the best variable [20].

### 2.7. Western Blot

Total proteins were extracted using a total protein extraction kit (Beyotime, China). Western blot was conducted based on the standardized process (10% SDS-PAGE gel). The primary antibody of CALCRL (1 : 2000) and GAPDH (1 : 50000) was purchased from Proteintech.

### 2.8. Statistical Analysis

This research was analyzed using R software version 4.2.1. Two-sided *P* values < 0.05 were considered statistically significant. For continuous variables with normal distributions, an independent *t*-test was applied, and for continuous variables with skewed distributions, a Wilcoxon rank-sum test was conducted. The study of differentially expressed genes (DEGs) was conducted using the limma program with the criteria |logFC| > 1 and *P* < 0.05.

## 3. Results

### 3.1. Immune Term Quantification

**Figure 1:**
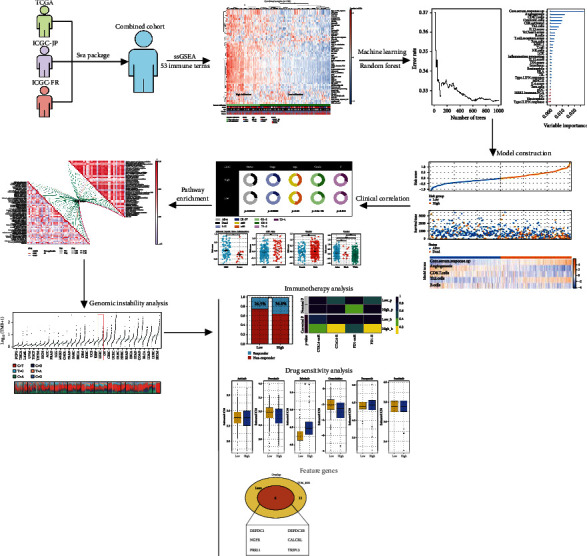
The flowchart of the whole study.

**Figure 2:**
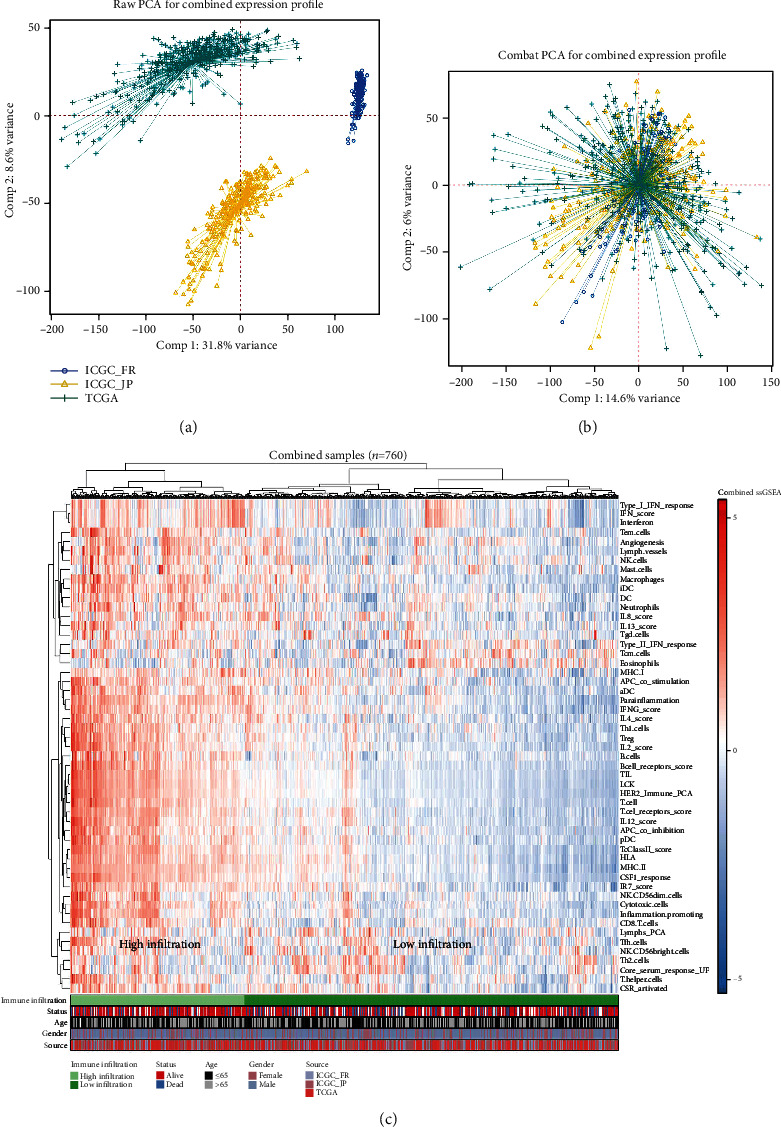
Quantification of 53 immune terms based on the ssGSEA algorithm. Notes: (a) three LIHC cohorts were selected for our analysis, including TCGA-LIHC, ICGC-FR, and ICGC-JP cohorts; (b) the combat function in sva package was utilized for data integration and batch difference reduction; and (c) 53 immune elements were measured using ssGSEA.

Figure 1 displays the flowchart of the entire investigation. Three distinct liver cancer cohorts, TCGA-LIHC, ICGC-JP, and ICGC-FR, were chosen for our research (Figure 2(a)). The sva package was employed to combine data and decrease the batch effect. Then, a significant batch impact decline was noted (Figure 2(b)). The ssGSEA method was employed in the pooled cohorts to quantify 53 immunological elements (Figure 2(c)).

### 3.2. Prognosis Model Construction

**Figure 3:**
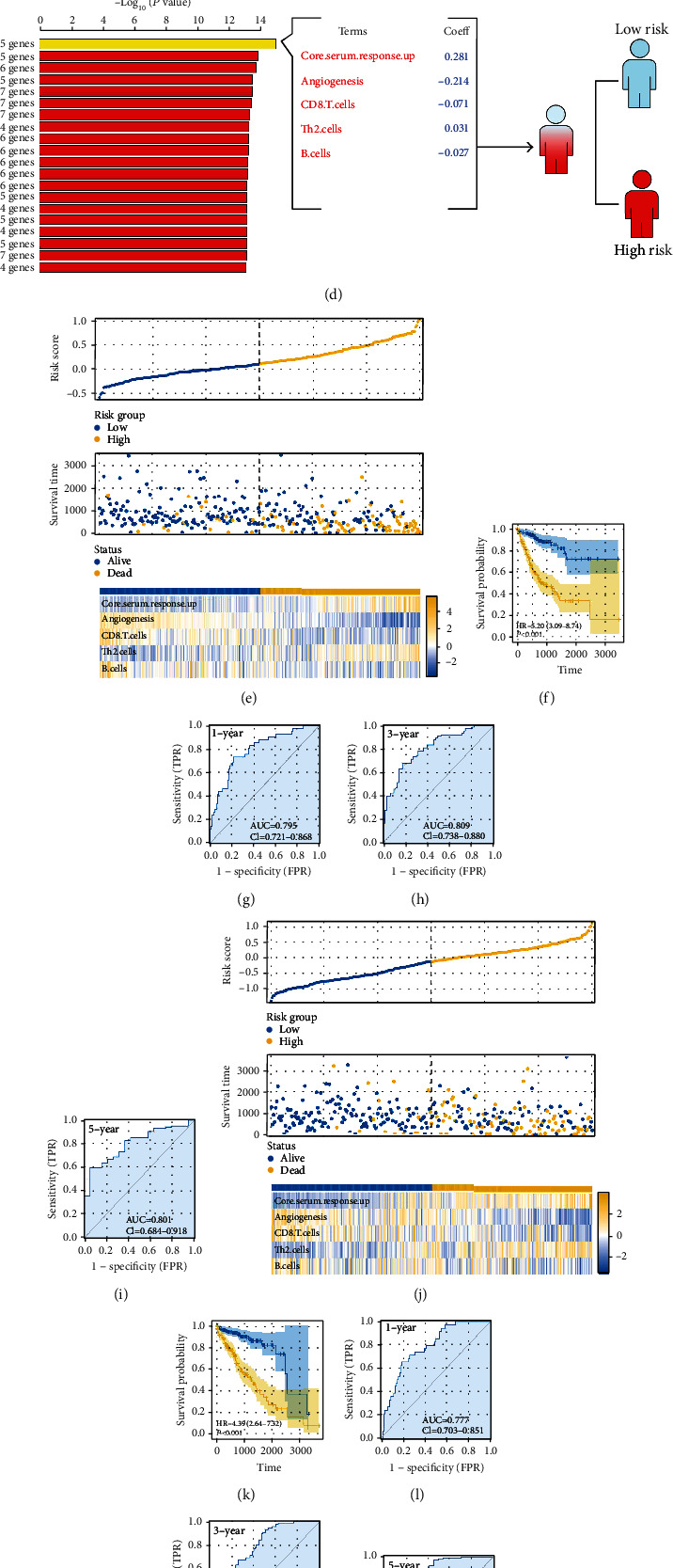
Prognosis model construction. Notes: (a) univariate Cox regression analysis was utilized to find prognosis-related elements with a significance level of *P* < 0.05; (b, c) the random forest technique was used for dimensionality reduction; (d) five elements, including CORE.SERUM.RESPONSE.UP, angiogenesis, CD8.T.cells, Th2.cells, and B.cells, were selected for model creation; (e) the summary of our model in the training cohort; (f) the KM curves indicated that high-risk patients may have a poorer outcome (training cohort); (g–i) ROC curves for one, three, and five years (training cohort); (j) the summary of our model in the validation cohorts; (k) the KM curves indicated that high-risk patients may have a poorer outcome (validation cohort); and (l–n) ROC curves for one, three, and five years (validation cohort).

First, the patients were randomly divided into training and validation cohorts according to the 1 : 1 ratio. Based on the 53 immunological elements, a univariate Cox regression analysis was conducted to find the prognosis-related variables with *P* < 0.05 (Figure 3(a) and Table 4). The random forest approach was then used to reduce the dimensionality, and the top ten important terms were CORE.SERUM.RESPONSE.UP, angiogenesis, CD8.T.cells, Cytotoxic.cells, CSR.activated, Th2.cells, IL13.score, TcClassII.score, B.cells, and T.cells.receptors.score (Figures 3(b) and 3(c)). Through multivariate Cox regression analysis and permutations, the five immune terms were used for prognosis model construction, including CORE.SERUM.RESPONSE.UP, angiogenesis, CD8.T.cells, Th2.cells, and B.cells (Figure 3(d)). The risk score was calculated with the formula of “Risk score = CORE.SERUM.RESPONSE.UP ^∗^ 0.281 + angiogenesis ^∗^ -0.214 + CD8.T.cells ^∗^ -0.071 + Th2.cells ^∗^ 0.031 + B.cells ^∗^ -0.027”. The overview of our model (training group) was shown in Figure 3(e), whereas a greater number of fatalities were seen in the high-risk group. The KM survival curve revealed that high-risk individuals tend to have a poorer outcome (Figure 3(f), HR = 5.20, *P* < 0.001, and concordance index = 0.893). ROC curves for 1-, 3-, and 5-year patients demonstrated a high predictive accuracy (Figures 3(g)–3(i), 1-year: AUC = 0.795, 3-year: AUC = 0.809, and 5-year: AUC = 0.801). Also, in the validation group, the same trend was observed (Figure 3(j)). KM survival curves indicated that the high-risk patients might have a worse prognosis performance (Figure 3(k), HR = 4.39, *P* < 0.001, and concordance index = 0.714). Meanwhile, the performances of ROC curves for 1-, 3-, and 5-year-old patients are still satisfactory (Figures 3(l)–3(n), 1-year: AUC = 0.777, 3-year: AUC = 0.763, and 5-year: AUC = 0.783).

### 3.3. Clinical Correlation Analysis

**Figure 4:**
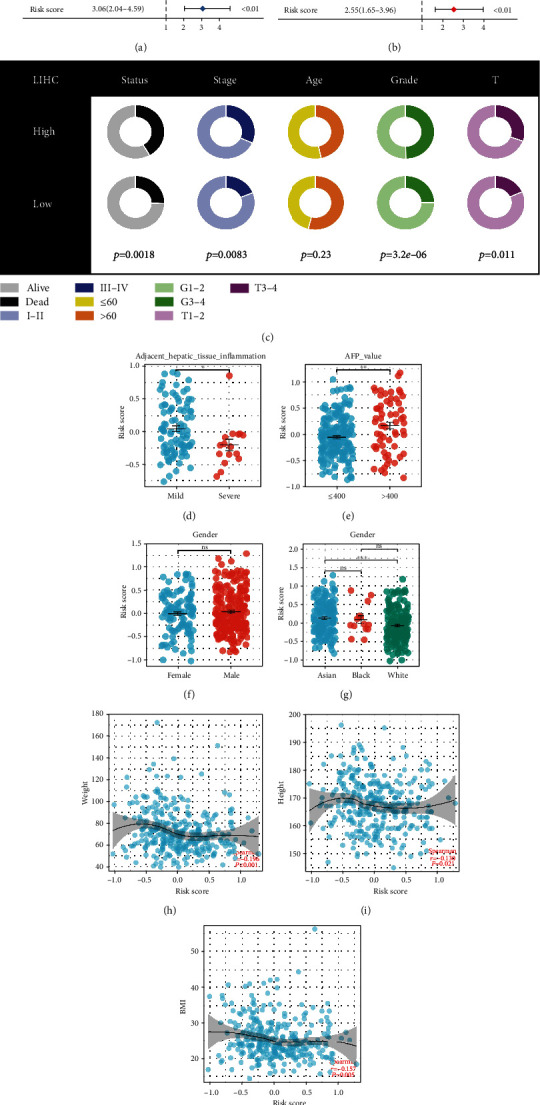
Clinical correlation of the riskscore. (a) Univariate Cox analysis of the riskscore as well as other clinical variables; (b) multivariate Cox analysis of riskscore and other clinical characteristics; (c) variations in clinical findings between high and low risk patients; (d–g) the riskscore difference in specific patients; and (h–j) the correlation of riskscore with patients height, weight, and BMI.

Furthermore, we explore the clinical correlation of our model. Univariate Cox regression and multivariate Cox regression analysis revealed because our model is not related to other clinical characteristics (Figures 4(a) and 4(b), univariate: HR = 3.06, *P* < 0.01; multivariate: HR = 2.55, *P* < 0.01). Clinical correlation analysis showed that high-risk patients could possess more aggressive clinical characteristics, such as clinical stage, grade, and T classification (Figure 4(c)). Interestingly, we found that the patients with mild adjacent hepatic tissue inflammation might have a higher risk score compared to the severe group (Figure 4(d)). Moreover, the patients with AFP > 400 ng/ml had a higher risk score than those with AFP < 400 ng/ml (Figure 4(e)). No significant difference was found in different gender patients. Asian populations might have a higher risk score than White populations (Figure 4(f)). A negative correlation was found between height, weight, and BMI (Figures 4(h)–4(j), weight, *R* = −0.196, *P* < 0.001, height, *R* = −0.130, *P* = 0.021, BMI, *R* = −0.157, *P* = 0.005).

### 3.4. Pathway Enrichment Analysis

**Figure 5:**
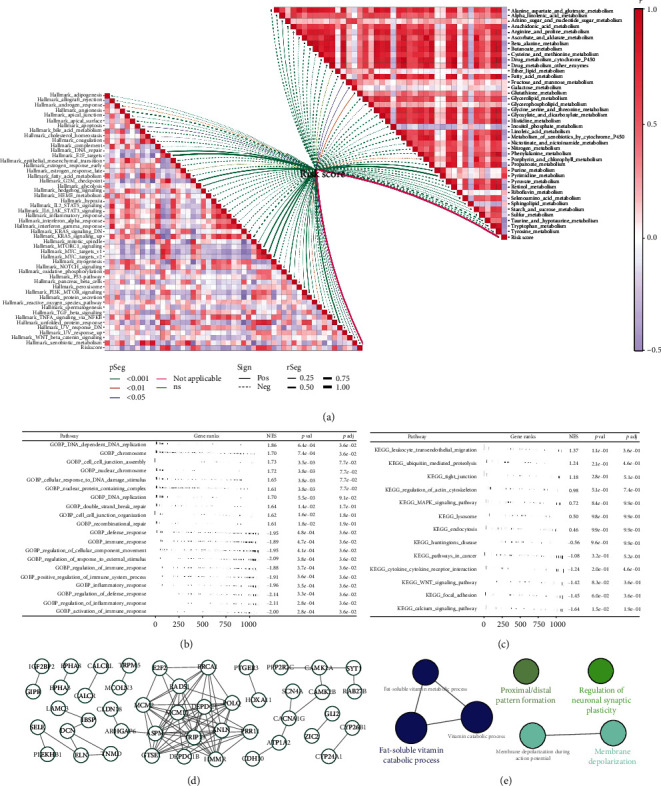
Pathway enrichment analysis. (a) GSVA analysis indicated that the correlation between riskscore and Hallmark and metabolism pathways; (b, c) GSEA analysis of GO and KEGG pathways; (d) the PPI network of the DEGs between high- and low-risk group; and (e) ClueGO analysis of the nodes.

We investigated the biological differences between high-and low-risk patients. For Hallmark pathways, we found that risk score was positively correlated with DNA repair, E2F targets, G2M checkpoints, HEDGEHOG signaling, mTORC1 signaling, and MYC targets (Figure 5(a)). For metabolism-related pathways, we observed that riskscore was positively correlated with purine and pyrimidine metabolism (Figure 5(a)). The GSEA method illustrated that in the high-risk group, the terms of DNA-dependent DNA replication, chromosome, cell-cell junction assembly, nuclear chromosome, leukocyte transendothelial migration, ubiquitin-mediated proteolysis, tight junction, actin cytoskeleton regulation, and MAPK signaling were upregulated (Figures 5(b) and 5(c)). Also, based on the DEGs discovered between high- and low-risk patients, a network of protein-protein linkages was developed (Figure 5(d)). ClueGO analysis revealed that these nodes were mainly enriched in fat-soluble vitamin catabolic process, proximal/distal pattern formation, regulation of neuronal synaptic plasticity, and membrane depolarization (Figure 5(e)).

### 3.5. Genomic Instability Analysis

**Figure 6:**
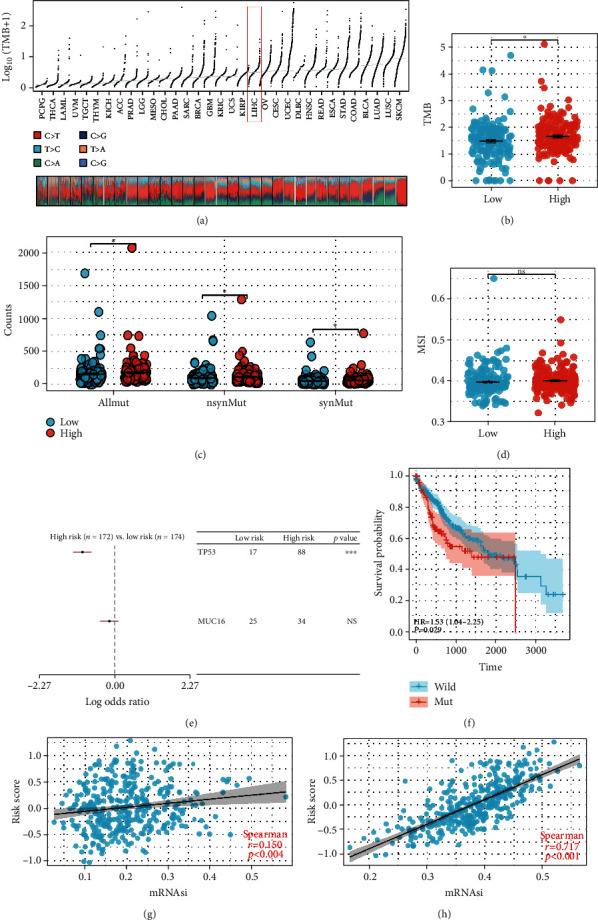
Genomic instability analysis. (a) A summary of the distribution of TMB in TCGA pan-cancer; (b) comparison of TMB between high-risk and low-risk patients; (c) the disparity between high-risk and low-risk patients' mutant count, nonsynonymous mutation count, and synonymous mutation count; (d) the MSI distinction between patients at high and low risk; (e) TP53 and MUC16 were the most frequently mutated genes comparing patients at high and low risk; (f) KM survival curve of the TP53 wild and mut patients; and (g, h) the correlation of riskscore with tumor stemness index.

The genomic feature can also affect the tumor biological process. Genetic variations are between high- and low-risk patients. Mutation information was obtained from the TCGA database (Figure 6(a)). The outcome indicated that high-risk patients may possess a greater TMB score (Figure 6(b)). Meanwhile, we found that all mutant counts, synonymous mutation counts, and nonsynonymous mutation counts were elevated in patients at high risk (Figure 6(c)). No significant variation in the MSI score was observed between high- and low-risk patients (Figure 6(d)). Moreover, we discovered that TP53 was the gene greatest substantially mutated across patients at high and low risk (Figure 6(e)). The KM curve indicated that patients with TP53 mutations may suffer a poorer outcome (Figure 6(f)). In addition, a positive significant correlation was identified between risk score and tumor stemness index (Figures 6(g) and 6(h), mDNAsi, *R* = 0.150, *P* = 0.004; mRNAsi, *R* = 0.71, *P* < 0.001).

### 3.6. Immunotherapy and Drug Sensitivity Analysis

**Figure 7:**
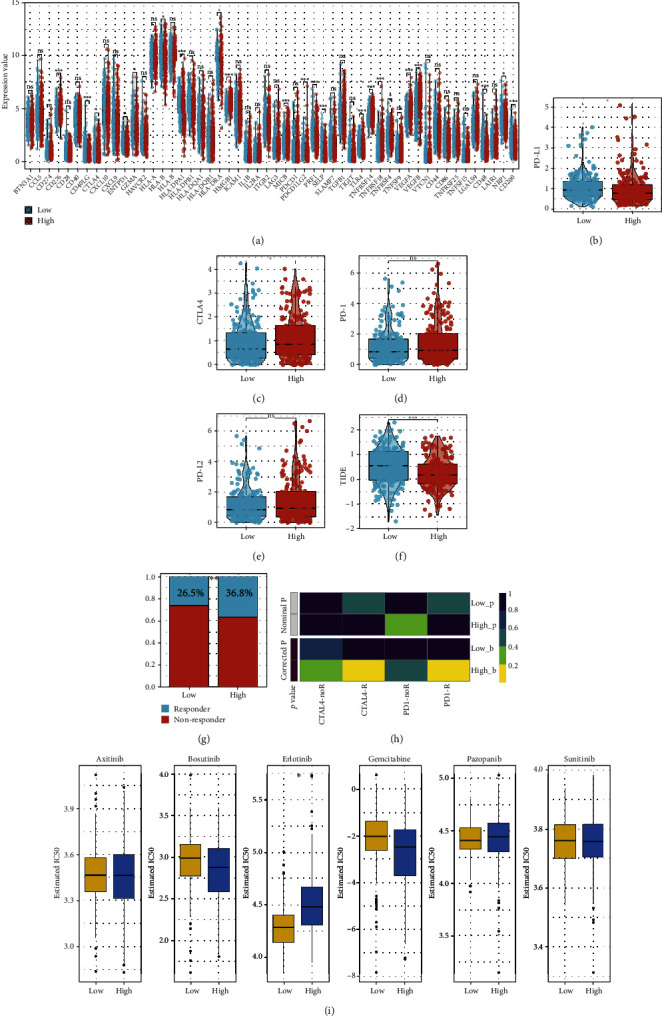
The risk score was associated with the sensibility to immunotherapy and chemotherapy. (a) Several disparities in immunological checkpoints between high- and low-risk groups; (b–e) variations in PD-L1, CTLA4, PD-1, and PD-L2 between high- and low-risk patients; (f, g) High-risk patients may have a lower TIDE score and a higher percentage of immunotherapy responders; (h) high-risk individuals may respond better to CTLA4 and PD-1 treatment; and (i) low-risk patients may respond better to Erlotinib.

Immune checkpoint modules contribute substantially to the course of cancer; hence, we analyzed the correlation between the risk score and numerous checkpoint modules. Correlation analysis indicated significant variations between the high- and low-risk groups for various immune checkpoint modules, such as PD-L1 and CTLA-4 (Figures 7(a)–7(e)). TIDE research revealed that high-risk patients may show a lower TIDE score and a greater probability of immunotherapy responses (Figures 7(f) and 7(g)). The submap algorithm suggested that high-risk patients could be highly responsive to PD-1 and CTLA-4 therapy (Figure 7(h)). Analysis of drug responsiveness revealed that low-risk patients could be higher responsive to Erlotinib (Figure 7(i)).

### 3.7. Identification of the Characteristic Genes of the Risk Group

**Figure 8:**
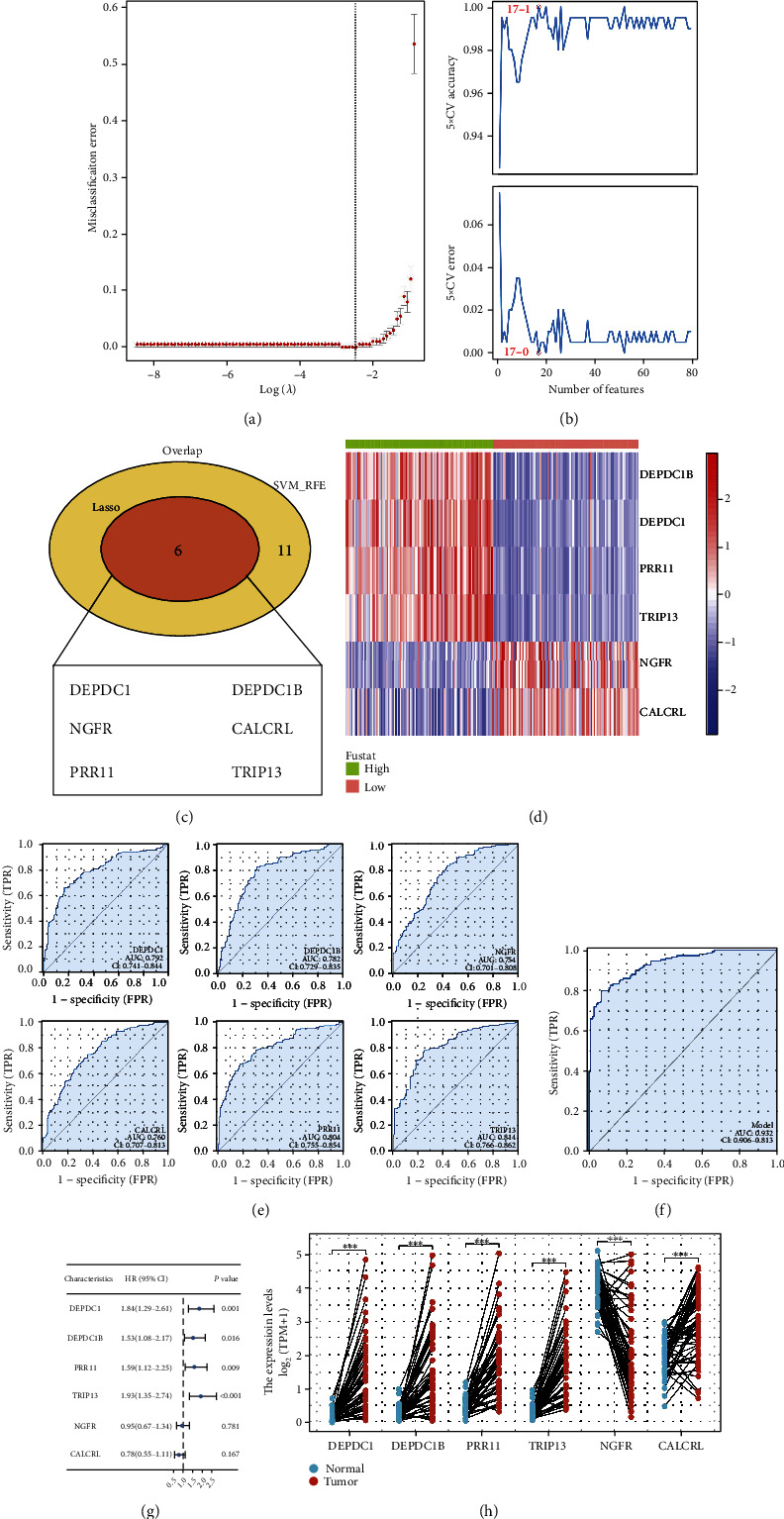
Identification of the characteristic genes of the risk group. Notes: (a) the LASSO regression algorithm was employed to determine the distinctive genes; (b) the SVM-RFE algorithm was utilized to determine the genes features; (c, d) DEPDC1, DEPDC1B, NGFR, CALCRL, PRR11, and TRIP13 were identified as the characteristic genes; (e) ROC curves were utilized to assess the prediction accuracy of characteristic genes on risk group (training cohort); (f) the logistic regression model showed an extremely high prediction efficiency in the risk group (training cohort); (g) the prognosis effect of DEPDC1, DEPDC1B, NGFR, CALCRL, PRR11, and TRIP13; and (h) expression level of these six genes in tumor and normal tissue.

Considering the prognosis and therapy sensitivity difference between high- and low-risk patients, we try to identify the characteristic gene that could robustly indicate the risk group. Utilizing LASSO regression and the SVM-RFE technique, distinctive genes were identified (Figures 8(a) and 8(b)). The intersection of these two algorithms identified six genes, including DEPDC1, DEPDC1B, NGFR, CALCRL, PRR11, and TRIP13 (Figure 8(c) LASSO logistic regression: DEPDC1, DEPDC1B, NGFR, CALCRL, PRR11, and TRIP13; SVM-RFE: CALCRL, MCM10, SCN4A, NGFR, DEPDC1, PRR11, DEPDC1B, RAD51, ANLN, PYGM, CD5L, DCN, IYD, GLI2, TRIP13, TNMD, and SLC12A1). Among these genes, DEPDC1, DEPDC1B, PRR11, and TRIP13 were increased in the high-risk group, while NGFR and CALCRL were decreased (Figure 8(d)). All these characteristic genes showed great prediction efficiency in the patients' risk group (Figure 8(e), training cohort, DEPDC1, AUC = 0.792; DEPDC1B, AUC = 0.782; TRIP13, AUC = 0.814; CALCRL, AUC = 0.760; PRR11, AUC = 0.804; NGFR, AUC = 0.754). Logistic regression was performed to combine these genes with the formula of “0.3245 + 0.3252∗DEPDC1 + 0.3248∗DEPDC1B + −0.8162∗NGFR + −1.388∗CALCRL + 0.3101∗PRR11 + 1.8485∗TRIP13”, which showed extremely great prediction efficiency in patients risk group (Figure 8(f), AUC = 0.932). In the validation cohort, these characteristic genes also showed great prediction efficiency in the patients' risk group (Figure S1A–F, DEPDC1, AUC = 0.816; DEPDC1B, AUC = 0.766; TRIP13, AUC = 0.833; CALCRL, AUC = 0.711; PRR11, AUC = 0.820; NGFR, AUC = 0.716), as well as the logistic regression model (Figure S1G, AUC = 0.896). Among these characteristic genes, DEPDC1, DEPDC1B, PRR11, and TRIP13 were risk factors for liver cancer patients (Figure 8(g)). Meanwhile, DEPDC1, DEPDC1B, CALCRL, PRR11, and TRIP13 were significantly upregulated in liver cancer tissue, yet NGFR was downregulated (Figure 8(h)). In liver cancer, the genes DEPDC1, DEPDC1B, PRR11, and TRIP13 have been explored in previous studies [21–24]. Therefore, we selected CALCRL for further validation. Western blot indicated that the protein level of CALCRL was upregulated in liver cancer tumor tissue (Figure S2).

## 4. Discussion

Liver cancer is still a serious public health concern worldwide [25]. Recently, researchers have focused on the immune microenvironment in liver cancer, which can significantly affect the progression of the disease. Thus, a comprehensive investigation of the liver cancer immune microenvironment can contribute to its diagnosis and therapy options.

In our study, we firstly quantified 53 immune terms in a combined cohort with large populations using the ssGSEA algorithm. Afterward, a prognostic model based on five immune elements (CORE.SERUM.RESPONSE.UP, angiogenesis, CD8.T.cells, Th2.cells, and B.cells) was established, which showed great prognosis prediction efficiency in both training and validation cohorts. Analysis of clinical correlations revealed that high-risk patients could possess higher clinical progression characteristics. Angiogenesis plays an important role in tumor metastasis and progression. From the comprehensive review conducted by Morse et al., abnormal angiogenesis in liver cancer often leads to poor prognosis and facilitated progression [26]. Wolf et al. found that the cross-talk between intrahepatic CD8+ T cells and NKT cells contributes to nonalcoholic steatohepatitis and liver cancer [27]. Xu et al. found that the Th2 response could affect liver fibrosis, which is a risk factor for liver cancer [28]. Garnelo et al. indicated that the interaction between tumor-infiltrating B cells and T cells significantly affects the progression of liver cancer [29].

Next, a pathway enrichment analysis was conducted to investigate the intrinsic biological variations between high-and low-risk patients. Analysis of genomic instability revealed that high-risk patients could possess a higher TMB score. The risk score showed a high positive correlation with the tumor stemness index. In addition, the TIDE outcome indicated that high-risk patients could be better responsive to immunotherapy, whereas low-risk patients could be better responsive to Erlotinib. Finally, six characteristic genes DEPDC1, DEPDC1B, NGFR, CALCRL, PRR11, and TRIP13 were identified for risk group prediction.

Pathway enrichment analysis showed that in high-risk patients, the Hallmark pathway of DNA repair, E2F targets, G2M checkpoints, Hedgehog signaling, mTORC1 signaling, and MYC targets were elevated. Malignant tumor cells have higher genomic damage compared to normal cells. Meanwhile, abnormal DNA repair procedures are difficult to meet the needs of DNA damage, therefore, leading to a more aggressive phenotype [30]. G2/M checkpoint is a critical phase in the cell cycle, which might directly affect cell proliferation [31]. Hedgehog signaling has been reported and extensively included in liver cancer progression. For instance, Gu et al. revealed that circular RNA circIPO11 could drive self-renewal and cancer progression of liver cancer through Hedgehog signaling, which might be an underlying therapeutic target [32]. In addition, Wu et al. discovered that CK2 might enhance stemness and chemotherapy resistance via the Hedgehog signaling pathway in liver cancer [33]. These findings suggested that patients in the high-risk category may have a higher level of activity in the aforementioned pathways, thus resulting in a poor prognosis.

Genomic analysis revealed that high-risk patients might have a greater level of genomic instability. In cancer, genomic instability is a prominent feature, which is also a key marker for separating cancerous cells from normal ones [34]. Genomic instability is defined as the increase in mutation frequency in the genome and its potential source is the sum of defects in the DNA damage and repair pathway, which could lead to uncontrolled proliferation of cancer cells [34]. In the pathway enrichment analysis, we have found the abnormal activity of DNA repair in high-risk patients. Therefore, higher genomic instability in high-risk patients seems to be reasonable. Moreover, a strong correlation was observed between the risk score and tumor stemness. Liver cancer has a high recurrence rate, making it one of the most highly deadly malignant tumors, partly due to the existence of cancer stem cells (CSC) [35]. Tumor stemness could also affect the immune microenvironment of liver cancer. For example, M2 TAMs could promote the expression of stemness proteins [36]. Another aspect, the CXCL11, a cytokine involved in the recruitment of activated T cells to inflammatory sites, was found significantly affect the stemness genes, sphere formation, and tumorigenicity in liver cancer [37].

Immunotherapy and chemotherapy are the best choices for the advanced liver cancer stage [4]. Low-risk patients could be higher responsive to Erlotinib, whereas high-risk patients could be more responsive to PD-1 and CTLA-4 treatment. According to the LASSO regression and SVM-RFE algorithm, we identified six characteristic genes, including DEPDC1, DEPDC1B, NGFR, CALCRL, PRR11 and TRIP13. The logistic regression model showed an extremely great prediction efficiency in the patients risk group. In the clinical application, measuring the expression level of these six genes might accurately identify the risk group of a patient, which might have the potential to guide the prognosis and therapy options of liver cancer patients.

Although our study was performed based on high-quality bioinformatics analysis, some limitations should be noticed. First, the populations included in our study were mainly White populations. Therefore, the underlying race bias might reduce the credibility of our conclusion. Second, some specific clinical features, for example, laboratory examination, lifestyle, and other basic diseases were not provided in most patients. However, our approach proved an excellent pattern for identifying liver cancer patients' survival time as well as their immunotherapy and chemotherapy responsiveness.

## Figures and Tables

**Table 1:** Baseline information of the patients in TCGA-LIHC.

Features	Numbers	Percentage (%)
Age		
≤65	235	62.3
>65	141	37.4
Unknown	1	0.3
Gender		
Female	122	32.4
Male	255	67.6
Grade		
G1	55	14.6
G2	180	47.7
G3	124	32.9
G4	13	3.4
Unknown	5	1.3
Stage		
Stage I	175	46.4
Stage II	87	23.1
Stage III	86	22.8
Stage IV	5	1.3
Unknown	24	6.4
T-stage		
T1	185	49.1
T2	95	25.2
T3	81	21.5
T4	13	3.4
Unknown	3	0.8
N-stage		
N0	257	68.2
N1	4	1.1
Unknown	116	30.1
M-stage		
M0	272	72.1
M1	4	1.1
Unknown	101	26.8

**Table 2:** Baseline information of the patients in ICGC-FR.

Features	Numbers	Percentage (%)
Age		
≤65	205	55.6
>65	164	44.4
Gender		
Female	76	20.6
Male	293	79.4
T-stage		
T1	54	14.6
T2	65	17.6
T3	40	10.8
T4	1	0.3
Unknown	209	56.6
N-stage		
N0	160	43.4
Unknown	209	56.6
M-stage		
M0	159	43.1
M1	1	0.3
Unknown	209	56.6

**Table 3:** Baseline information of the patients in ICGC-JP.

Features	Numbers	Percentage (%)
Age		
≤65	98	37.7
>65	162	62.3
Gender		
Female	68	26.2
Male	192	73.8
Stage		
Stage I	40	15.4
Stage II	117	45.0
Stage III	80	30.8
Stage IV	23	8.8

**Table 4:** The terms significantly related to prognosis based on the univariate Cox regression.

Gene	HR	*P* value	Lower	Upper
CORE.SERUM.RESPONSE.UP	1.733	<0.001	1.496	2.007
Th2.cells	1.478	<0.001	1.274	1.714
CD8.T.cells	0.676	<0.001	0.572	0.800
Cytotoxic.cells	0.687	<0.001	0.583	0.809
Angiogenesis	0.724	<0.001	0.629	0.834
CSR.Activated	1.347	<0.001	1.158	1.567
IL13.score	0.759	0.001	0.648	0.891
B.cells	0.767	0.001	0.656	0.896
T.cell	0.783	0.003	0.667	0.920
Type.I.IFN.Reponse	0.804	0.005	0.691	0.935
TcClassII.score	0.797	0.005	0.680	0.933
TIL	0.795	0.006	0.676	0.936
LCK	0.803	0.007	0.684	0.942
NK.cells	0.815	0.009	0.698	0.951
Type.II.IFN.Reponse	0.828	0.009	0.718	0.955
MHC.I	0.829	0.011	0.717	0.959
Neutrophils	0.823	0.014	0.704	0.961
T.cell.receptors.score	0.834	0.018	0.718	0.969
DC	0.832	0.018	0.715	0.970
MHC.II	0.833	0.019	0.716	0.971
Tcm.cells	0.837	0.019	0.720	0.972
IFN.score	0.835	0.021	0.717	0.974
HLA	0.840	0.027	0.720	0.980
Inflammation.promoting	0.838	0.029	0.715	0.983
Interferon	0.845	0.031	0.726	0.985
Eosinophils	0.851	0.033	0.733	0.987
IL12.score	0.844	0.038	0.719	0.990
Tem.cells	0.852	0.040	0.731	0.992
pDC	0.853	0.043	0.731	0.995
HER2.Immune.PCA	0.849	0.045	0.724	0.997
IL4.score	0.857	0.049	0.735	0.999

## Data Availability

Based on reasonable request, data are available from the corresponding author.

## Supplementary Materials

Supplementary 1 Figure S1: the prediction accuracy of characteristic genes on risk group in validation cohort. Notes: (a–g): ROC curves of characteristic genes.

Supplementary 2 Figure S2: Western blot of CALCRL.
