# Supplementary figures and images for: Identification of a Five Immune Term Signature for Prognosis and Therapy Options (Immunotherapy versus Targeted Therapy) for Patients with Hepatocellular Carcinoma
Source: Comput Math Methods Med. 2023 Feb 2;2023:8958962. doi: 10.1155/2023/8958962 (PMC9918845; doi:10.1155/2023/8958962)

**Figure S1. The prediction accuracy of characteristic genes on risk group in validation cohort**

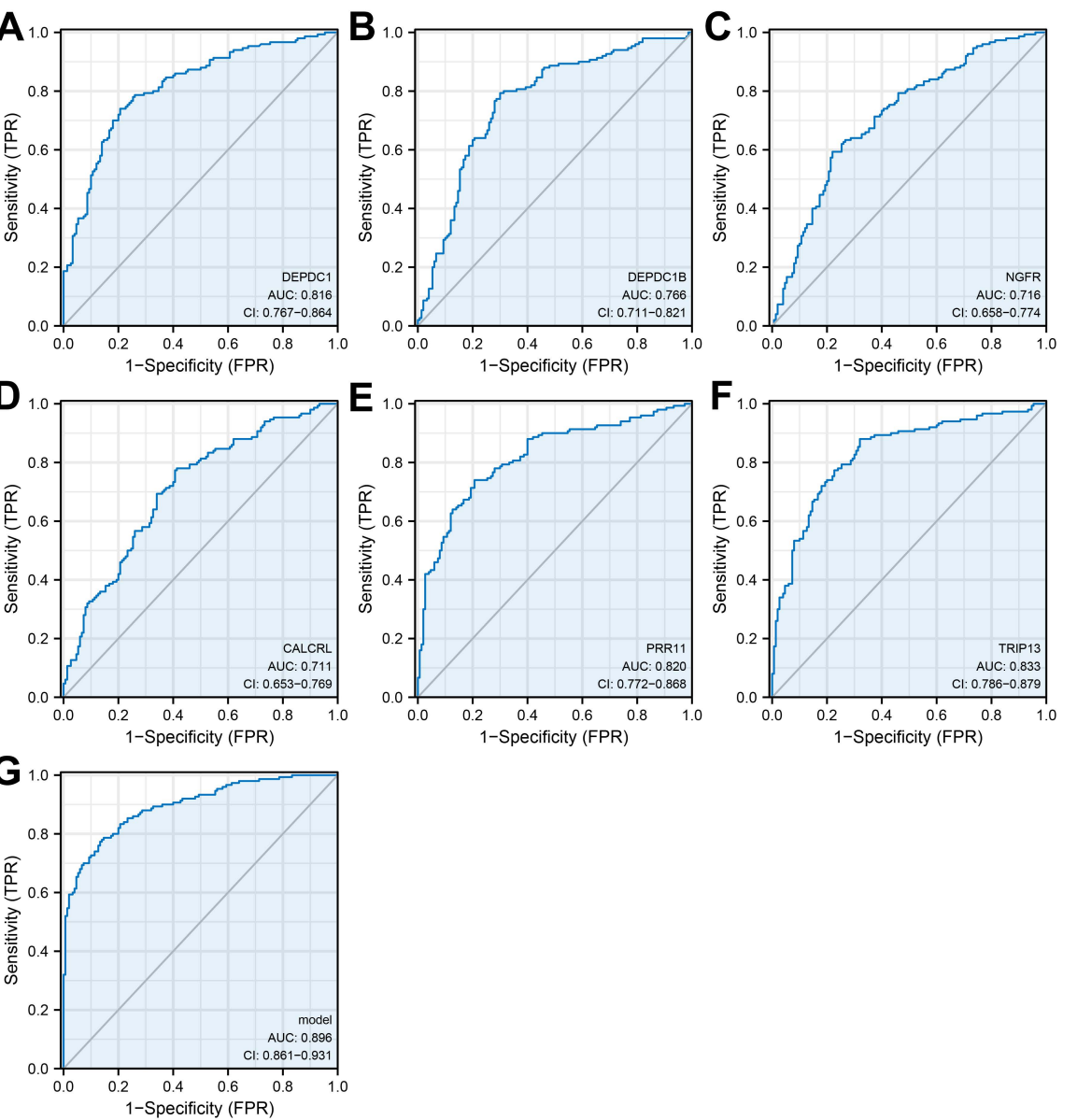

Supplement: Supplementary 1 — Figure S1: the prediction accuracy of characteristic genes on risk group in validation cohort. Notes: (a–g): ROC curves of characteristic genes. [file 8958962.f1.pdf]

**Figure S2. Western blot of CALCRL**

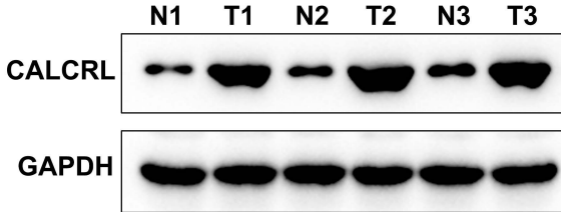

Supplement: Supplementary 2 — Figure S2: Western blot of CALCRL. [file 8958962.f2.pdf]
